# 

**Published:** 1951-10

**Authors:** 


					V \
I
PUBLISHED UNDER THE AUSPICES OF THE BRISTOL MEDICO-CHIRURGICAL SOCIETY
THE BRISTOL
MEDICO - CHIRURGICAL
JOURNAL
A Journal of the Medical Sciences for the
WEST OF ENGLAND
AND SOUTH WALES
Editor:
E. WATSON-WILLIAMS, M.C., M.D., Ch.M., F.R.C.S.
with whom are associated
G. E. F. SUTTON, M.C., M.D., F.R.C.P.
J. H. MIDDLEMISS, M.D., D.M.R.D., F.F.R.
N. S. CRAIG, M.C., M.D., Assistant Editor.
Local Correspondents :
Bath?A. D. BATEMAN, O.B.E., F.R.C.S. f
Exeter?L. N. JACKSON, M.C., D.M.
Gloucester?R. F. JARRETT, M.B., M.R.C.P.
Newport?J. L. D. WILLIAMS, M.B., F.R.C.S. '
Plymouth?C. R. CROFT, D.M., M.R.C.P. Taunton?Dr. M. McA
Torquay?Dr. A. ROBINSON THOMAS, D.M.R.E.
Truro?Dr. F. D. M. HOCKING, F.R.LC. Yeovil?J. A. VERE NICOLL,
BRISTOL I
J. W. ARROWSMITH LTD., QUAY STREET AND SMALL STREET
V0l. LXVm. No. 248. Price > FOUR SHILLINGS net.
OCTOBER ' 1951

				

